# Bullying and Cyberbullying among Italian Adolescents: The Influence of Psychosocial Factors on Violent Behaviours

**DOI:** 10.3390/ijerph18041558

**Published:** 2021-02-06

**Authors:** Antonio Tintori, Giulia Ciancimino, Giorgio Giovanelli, Loredana Cerbara

**Affiliations:** 1 Institute for Research on Population and Social Policies of the National Research Council of Italy (CNR-IRPPS), 00185 Rome, Italy; giulia.ciancimino@irpps.cnr.it (G.C.); loredana.cerbara@irpps.cnr.it (L.C.); 2 Department of Social Sciences and Economics, Sapienza University of Rome, 00185 Roma, Italy; giorgio.giovanelli@uniroma1.it

**Keywords:** bullying, cyberbullying, adolescents, social deviance, violent behaviours, psychosocial factors, social conditioning, stereotypes, self-esteem, survey

## Abstract

Background: The study of adolescents’ behaviours and attitudes is crucial to define interventions for the containment of deviance and social discomfort. New ways of social interaction are crystallising violent behaviours which are moving more than ever on a virtual sphere. Bullying and cyberbullying share a common behavioural matrix that has been outlined through specific environmental and individual characteristics. Methods: A survey carried out in Italy in 2019 on a statistical sample of 3273 students highlighted the influence of several social and individual variables on deviant phenomena. Risk and protective factors in relation to the probability of involvement in bullying and cyberbullying have been shown through a bivariate analysis and a binary logistic regression model. Results: The study shows that presence of stereotypes and social prejudices, tolerance to violence and high levels of self-esteem have resulted as the main risk factors. On the other hand, low levels of tolerance related to the consumption of alcohol and drugs, high levels of trust towards family and friends and being female have been identified as protective factors. Conclusions: This research confirms the validity of several theories on bullying and cyberbullying phenomena. Furthermore, it identifies specific risk and protective factors and their influence on deviant behaviours, with a focus on environmental characteristics which appear as the key field of work to enhance adolescents’ well-being.

## 1. Introduction and Objectives

In 2014, in Italy, over 50% of adolescents complained of being subjected to violent actions; in particular, 19.8% of boys and girls between 11 and 19 years old have been victims of aggressive behaviours both physical and verbal [1]. These behaviours may trace back to the bullying phenomenon, which includes “*aggressive, intentional acts carried out by a group or an individual repeatedly and overtime against a victim who cannot easily defend him or herself*” ([2], p.48). Bullying acts may happen through physical violence, social manipulation—indirect bullying—or by damaging relations—relational bullying [3]. The existence of an asymmetric relationship among subjects involved unites these different forms [4]. The perception of power asymmetry lies at the bottom of the relationship between victims and bullies, a relation that is characterised by unequal and coercive power [5]. 

Bullies and victims are the main categories of the bullying phenomenon, representing the two opposites in the violent relationship among peers. However, other figures come abreast of them as the bullies/victims, a mixed category that comprises bullies who have been victims of bullying [6,7]. Moreover, episodes of violence occur at the presence of bystanders, who are seen as spectators not directly get involved with bullying act [8]. Therefore, bullying appears as a multiform relational phenomenon expression of socio-relational distress which, at school, assumes a group dimension [9]; in particular, bullying acts represent the most common form of violence among children and adolescents [10]. School, then, is the most critical place where it is possible to study these phenomena and to realize interventions aimed at prevention of antisocial behaviours. In order to achieve this policy aim, it is important to intercept the phenomenon at every school level, with a particular attention to secondary schools where bullying seems to be less perceived by students. In this way, several studies show that the phenomenon seems to decrease with age [11]. However, this decrease seems to depend on two reasons. On the one hand, younger students are more at risk of being victims; on the other, they tend to “*perceive and define behaviour patterns as bullying that originally hadn’t been defined as bullying from the researchers*” ([12], p. 272). Bullying doesn’t express oneself only with the physical presence of the involved subjects. When aggressive behaviour occurs through technological devices, it assumes the denomination of cyberbullying [13]. Although internet-mediated bullying reproduces the bully/victim relationship, cyberbullying has to be considered as “*a unique phenomenon, distinguished from traditional bullying by a range of factors including the speed at which information is distributed, permanence of material and availability of victims*” [14]. Thus, cyberbullying offers a new virtual space to express violent behaviours, which are favoured by the anonymity of the Internet [15]. 

However, scientific literature highlighted that bullying and cyberbullying share a common behaviour pattern. Deviant and violent behaviours are associated with a series of individual and social variables which profile both the figures of bully and victim. Bullies, for instance, adopt aggressive and dominance behaviours [7,16] and have a low consideration for their victims’ suffering [17]. As Hawker and Boulton claim: “*depression and loneliness emerged as the strongest predictors of peer-reported victimization*” ([18], p. 452). Besides, feelings of insecurity and anxiety are two recurrent elements among victims of physical, verbal, and indirect bullying [7,19]. In other words, victims are described as isolated subjects with anxiety and depression problems, that may paradoxically find in online bullying a place where they revenge themself of offline bullying [20]. 

Among the psychological variables linked to bullying and cyberbullying phenomena, the concept of self-esteem (SE) deserves a deepening. Even if “*bullying does not seem to be connected to either very high or especially low (self-reported) SE*” ([21], p. 1270), self-perception provides some significant elements to better understand bullying and cyberbullying phenomena. Egan and Perry consider low self-esteem as a factor that may positively contribute to victimisation in subjects who have difficulty in claiming their needs and standing up for themselves during conflicts [22]. If the relation between low levels of self-esteem and bullying victimisation is widely accepted by literature, the relation between self-esteem and the perpetration of violent behaviours is still under debate. On the one hand, some authors [23] suggest that bullies have lower self-esteem than children and adolescents who do not bully. Results show that high self-esteem protects children and adolescents from involvement in bullying. Therefore, the low SE seems to characterise both victims and bullies in traditional bullying. This is confirmed also by studies on cyberbullying which associate “*significantly lower levels of self-esteem*” to bullies and victims ([24], p. 619). Conversely, other studies [25,26,27] associate violent behaviours to high levels of self-esteem, arguing that narcissism, which is linked to a high level of self-esteem, leads to aggressive behaviours.

A significant difference between online and offline violent behaviours concerns the distinction between bullying and cyberbullying perpetrators. Those who have high levels of self-esteem “*are more comfortable with face to face confrontations […] those with low self-esteem may be drawn to the relative safety and anonymity of the online environment*” ([14], p. 258). Therefore, if the literature provides dissimilar explanatory keys on this aspect, the idea that Internet-mediated bullying may favour deviant behaviours in subjects with low self-esteem who find a sort of protection in internet anonymity appears more consolidated.

Scientific literature highlights also the role of social variables, like the social status, social stereotypes and the relation with parents, as predictors of bullying and victimisation process. Sentse, Kretschmer and Salmivalli carried out significant researches on the relationship between social status and bullying in terms of peer acceptance and rejections. The study showed that subjects with low social status become easy targets for bullies, producing a vicious circle in which “*victimisation contributed to lower sociometric status (lower peer acceptance or higher peer rejection)*” ([28], p.672). At the same time, those who adopt aggressive behaviours may be connected to a high social status. According to Prinstein and Cillessen “*aggressive adolescents are generally high status, highly visible members of the social milieu who are not necessarily well-liked*” ([29], p. 334). However, social status is not equally shared among bullies. Following this perspective, Brighi et al. distinguish between two types of bullies—marginalized and connected—who possess different social skills and possibilities [30]. Social stereotypes are critical factors in victimization processes. First of all, stereotypes of race and ethnicity affect the possibility of becoming victims of bullying. This effect is amplified for students who break the stereotypes “*the violation of racial and ethnic stereotypes may created bullying victimization among racial and ethnic minority youth*” ([31], p. 559). Secondly, stereotyped gender attributions are predictors of bullying behaviors, especially in the form of homophobic bullying [32]. As mentioned above, the relationship between children and parents is a strong predictor of both bullying and victimisation behaviours. Georgiou and Stavrinides show that conflicts with parents are associated with aggressive behaviours at school [33]. The parenting style and the relationship’s quality may play a key role in the development or prevention of deviant behaviours. In particular, an aggressive and absent parenting style foster the participation in bullying acts, while a high involvement of parents and frequent communication with children distance the possibility to become bullies [34]. Also, an excessively protective maternal style may produce insecurity in children in favour of their victimisation [7]. 

The aim of this work is to study the phenomena of bullying and cyberbullying from a psychosocial perspective, investigating potential risk and protective factors. We hipothesised that the involvement in violent behaviours has strong links with self-perception and attitudes towards others at the same time. For this reason we have taken into consideration simultaneously individual variables, which have been considered relevant by literature, such as the one of self-esteem, and social variables as parental cultural status, vertical and horizontal trust, but also the adherence to stereotypes and discriminatory attitudes towards social diversity. The hypothesis test has been carried out using indicators of tolerance of bullying and cyberbullying, analysing the adherence to social stereotypies, but also considering other variables concerning the recognition of violent behaviours, the set of values owned by respondents, the propensity to alcohol and drugs consumption, the parental cultural status and the level of trust place in family, friends and teachers.

## 2. Materials and Methods 

This research is based on a survey carried out in Italy during 2019 on a sample of students attending upper secondary school. Selected schools were located in 12 cities which are representative of the four main national macro-areas (North-West, North-East, Center, South). For each city, three schools (one high school, one technical college and one vocational college, for a total of 36 schools) were randomly selected from the lists of schools of the Italian Ministry of University and Research (MUR). Within each school sampled, five classes were randomly selected—one for each year of school. Therefore, the age of the sample is between 13 and 22 years and the average, which coincides with the modal value, is 16 years. The resulted sample is representative of Italian students attending the upper secondary school and amount to a total of 3273 students. The study was carried out through the submission of an electronic semi-structured questionnaire in the presence of at least one researcher from the research team. This method has been applied in order to achieve a high quality of collected data through the electronic questionnaire [35]. 

During the data analysis phase, great attention was paid to multiple dimensions of the phenomenon. Some of these dimensions have been used for the validation of the hypothesis provided in this article. The indicators used in this work are original. They have been tested during previous experiences of the working group and subjected to multidimensional scaling analysis (MSA) when possible in order to verify the dimensionality of the components of the indicators. This analysis, which is considered more effective than the calculation of known indices (for example by Cronbach’s alpha or Omega index [36,37]), allows to verify the effective one-dimensional nature of the components used for the calculation of the Rosenberg’s self-esteem scale. It is well-known in literature, but it has been also verified in the context of this research to confirm the effectiveness of the index construction. All the indices presented here are therefore one-dimensional in origin or preliminarily tested through MDA analysis, otherwise they have not been used. Specifically, the variables that have been used in this study are as follows:

- Tolerance of bullying and cyberbullying indicator. This is a dummy variable for the selection of respondents who were tolerant of bullying and cyberbullying; this is the target variable of this study because it is an indicator deriving from specific questions that allow to identify respondents who tolerate bullying and cyberbullying. It is a selective indicator, which has been built in order to select from the overall sample those respondents who are at risk to be involved in bullying and cyberbullying, as victims, perpetrators and bystanders. It should be noted that the choice not to submit a direct question to unmask the bullying or cyberbullying attitude was determined by the need to avoid a possible underestimation of the phenomena due to the social desirability bias. [38,39]. This indicator is a proxy of the involvement in bullying and cyberbullying roles.
- Tolerance of violent or discriminatory behaviours and attitudes indicator. It is a dummy variable for the selection of students who have stated that they are tolerant of sexting, dating violence, racism and homophobia. Even in this case, as with the bullying and cyberbullying tolerance indicator, we preferred not to submit a direct question. Therefore, this indicator is a proxy of the involvement in violent behaviours:
- *Parental cultural status*. This variable arises from the data collected on the educational qualifications of the parents of respondents. An overall variable has been calculated, starting from the 6 response modes within a range from 1—low parental cultural status—to 6—high parental cultural status.
- *Recognition of violent behaviours indicator*. This indicator was built on the basis of the occurrence of the recognition of certain forms of violence of both physical and psychological kind. The indicator has values ranging from 0—absence of violence recognition—to 7—high degree of violence recognition.
- *Propensity to consumption of alcohol and drugs indicator*. This variable summarises six dummy variables for the selection of respondents who are tolerant towards the consumption of alcohol and drugs. The values of the indicator range from 0—low propensity to consume—to 6—high propensity to consume.
- *Xenophobia indicator*. Prejudices linked to Italian geographical differences indicator.
- Homophobia indicator.

These are three indicators that follow the same logic of construction. They show the degree of adherence to stereotypes towards foreigners, populations from different geographical areas of Italy and homosexuals. A 4-point Likert scale has been used to measure the agreement of respondents ranging from strongly disagree to strongly agree. As with many investigations we have conducted previously, we have chosen not to use the neutral option of the traditional Likert scale. As shown by many studies, we have found that the presence of this option does not reduce the cases of false answers, but, on the contrary, increases the number of people who, due to cognitive effort or social desirability, avoid expressing their opinion [40,41,42,43]. All the variables have a variation range between 1—low adherence to stereotypes—and 4—high adherence to stereotypes.

- *Set of values indicator*. This indicator was built through two variables: the first was linked to activities carried out by respondents during their leisure time, while the second concerned the importance given by respondents to the values of democracy, honesty, social participation and culture.
- *Relational trust indicators*. These are two distinct indicators. The first summarises the level of trust that respondents place in their own parents, siblings and friends, while the second concerns the level of trust placed in school teachers and sport coaches. The results are two indicators which show 3 different level of trust: low, medium and high level.
- *Rosenberg self-esteem scale*. An indicator derived from the score obtained by the self-esteem responses (low self-esteem for scores up to 15, healthy self-esteem for scores between 16 and 25, high self-esteem for scores above 26).

A bivariate analysis has been carried out in order to verify the relation among the target Indicator of tolerance of bullying and cyberbullying, the abovementioned variables and other structural variables such as gender, age, citizenship, type of school attended and the Rosenberg self-esteem scale [44]. Furthermore, this study shows the results of a binary logistic regression model that uses the target variable to calculate the probability of belonging to the category at risk of involvement in bullying and cyberbullying based on certain characteristics. 

The model is:

(1) $$ln\left(OR\right)=a+b_{x}x_{i}$$

where *OR* is the Odds Ratio of the target variable, i.e., the ratio between the probability of belonging to the category at risk of involvement in violent behaviours and its complement to 1 (therefore a value greater than 1 indicates that the probability of belonging to this category is greater than the probability of not belonging); *a* and *b*_x_ are the coefficients of the model; *x*_i_ is the vector of variables involved in the model. In our case, the vector used is composed of variables at different levels of measurement. However, they are eligible in this kind of model. The variables which have been involved in the model 1 are the following: xenophobia, propensity to consumption of alcohol and drugs, trust towards parents, siblings and friends, trust towards school teachers and sports coaches, gender, citizenship, suffered acts of violence, Rosenberg self-esteem scale, tolerance of violent or discriminatory behaviours and attitudes.

## 3. Results

On the basis of the abovementioned tolerance of bullying and cyberbullying indicator, the results show that 7.4% of respondents are tolerant of bullying and cyberbullying. This group of subjects was composed of 81.8% males and 18.2% females. Among them, 39.3% attend a technical college, 36% a vocational college and the remaining 24.8% a high school. More specifically, the percentage of students who are tolerant of bullying and cyberbullying rises to 9% among foreigners, while stands at 7.2% among Italians.

If we look at the family background, among respondents with a low level of parental cultural status, the percentage of students who are tolerant of bullying and cyberbullying increases at 10.3%, while it decreases at 7.8% and 7.2% with medium and high levels of parental cultural status.

In relation to the self-esteem Rosenberg scale, the percentage of those who tolerate bullying and cyberbullying among respondents with a low level of self-esteem is 6.7%, standing at 6.8% among students with a normal level of self-esteem, while it reaches 10.1% with a high level of self-esteem. Therefore, according to analysed data, as highlighted by several authors [25,26,27], we can confirm the relation between the involvement in bullying and cyberbullying phenomena and high levels of self-esteem among adolescents.

In addition, analysing stereotyped attitudes of students towards foreigners and homosexuals, as adherence to stereotypes rises, so does the percentage of respondents who are tolerant of bullying and cyberbullying. Indeed, results show a linear trend: among students with a low level of adherence to xenophobic stereotypes there is a 3.8% of respondents who are tolerant of bullying and cyberbullying; this percentage increases with a medium and a high level of acceptance of stereotypes, respectively to 6.2% and 11.6%. Concerning the relation between homophobic stereotypes and tolerance of bullying and cyberbullying, with a high level of adherence to stereotypes the percentage of tolerance is 12.7%, while with medium e low levels of homophobia it decreases respectively to 4.9% and 2.8%. Furthermore, the adherence to gender stereotypes confirms this trend: the percentage of agreement to the item “at home it is the man who must have the lead” is 18.2% among students who are tolerant to bullying and cyberbullying; whilst the percentage of adherence to the item “ It is up to the man to maintain the family” is 13.3% (the survey was carried out in Italian. To help the reader these two items have been translated into English). Then, the findings confirm links between stigma and deviant attitudes, which can give rise to discrimination and violence, as suggested by many authors [32,45,46].

Furthermore, collected data show that tolerance of violent or discriminatory behaviours and attitudes is positively related to tolerance of bullying and cyberbullying: as tolerance of sexting, dating violence, racism and homophobia increases, the acceptance of bullying and cyberbullying rises. With a high level of tolerance of these variables the percentage of respondents who are tolerant of bullying and cyberbullying is 25.2%, it decreases to 6.7% with a medium level of tolerance of violent behaviours, falling to 0% among respondents with a low level of tolerance of violent behaviours.

On the other hand, the recognition of violent behaviours is inversely related to tolerance of bullying and cyberbullying: among respondents with a low level of recognition, 14.8% is tolerant of bullying and cyberbullying. This percentage decreases with medium and high levels of recognition, respectively to 6% and 3.3%.

Another relevant variable which influence bullying and cyberbullying is the role of personal values [47,48]. The analysis of the set of values of the students interviewed reveals that only 3.9% of respondents who give importance to values of democracy, honesty, social participation and culture, are tolerant of bullying and cyberbullying. In particular, the percentage of respondents embedding the abovementioned set of value is 30.2% with a low level of tolerance of bullying and cyberbullying, decreasing with medium ad high levels of tolerance respectively to 23.2% and 12.7%.

Furthermore, as mentioned above, some authors [33,34,49] have found a relation between the level of trust and attachment that adolescents place in their own parents, siblings and friends and the involvement in bullying and cyberbullying behaviours. Results show that with a low level of trust the percentage of tolerant subjects stands at 8.9%. It decreases as the level of trust increases, settling at 7.1% with a medium level of trust and at 6.7% among respondents with a high level of trust. On the other hand, regarding levels of trust in teachers and sport coaches, it shows that with low and medium levels of trust the percentage of tolerant of bullying and cyberbullying remains stable at 7.6%, while it decreases to 6.6% of respondents with a high level of trust.

Another relevant consideration concerns victims of bullying and cyberbullying. The percentage of students who state to have been victims of violent behaviours is 51.4%. This group of respondents is composed of 51.6% of females and 48.4% males. Among them, 39.6% attend a high school, 29.5% a vocational college and the remaining 30.9% a technical college. Within this specific group, the percentage of respondents who are tolerant of bullying and cyberbullying is 6.5% against the 8.3% of respondents among those who declared to have never been victims of violent behaviours. However, if we take a closer look at the gender composition of the victims’ group, we notice that the percentage of males who are tolerant of bullying and cyberbullying rises to 10.3%, while the percentage of females stands at 3%.

The relationship between victims of bullying and self-esteem has been analysed by many studies [22,50]. According to them, victims of bullying tend to have lower self-esteem than non-victims. Concerning the self-esteem Rosenberg scale, results show that 33.3% of victims has a low level of self-esteem against 15% of respondents with a low level of self-esteem who have never been victim of violence 54.9% of victims has a normal level of self-esteem against 57.7% of non-victims having the same level of self-esteem. The remaining 11.8% of victims has a high level of self-esteem against 27.1% of non-victims.

In order to better analyse the well-being of victims of violent behaviours in terms of self-esteem, it is necessary to distinguish between physical and virtual violence. Among respondents, 8.7% state to have been victims of cyberbullying against 21.7% who state to have been victims of physical violence from bullying strategies. Furthermore, looking at the self-esteem Rosenberg scale, 46.5% of students who were victims of cyberbullying has a low level of self-esteem against 31.9% of respondents who were victims of physical violent behaviours. 48.6% of victims of cyberbullying has a normal level of self-esteem against 55.1% of victims of physical violence from bullying strategies, while the remaining 4.9% of victims of cyberbullying has a high level of self-esteem against 13% of victims of physical violence. Therefore, as many authors have found out [24], results confirm the strong relation between cyberbullying victimisation and low level of self-esteem. Finally, we analysed the feeling of happiness among victims in comparison with the ones perceived by non-victims. Results show that among victims of physical violence 41.9% perceive low level of happiness, 42.5% have a medium level of happiness, whilst the remaining 15.7% have high levels of happiness. On the other hand, victims of cyberbullying have lower level of happiness: 50.4% have a low level of happiness, while 34.9% and 14.8% have respectively medium and high level of happiness. If we look at students who have never been victim of violence, we notice a higher level of perceived happiness: 26.2% and 47% have respectively low and medium levels of happiness, while 26.6% have a high level of happiness. According to scientific literature [7,19], bullying and, to a larger extent, cyberbullying are linked to the risk of higher levels of unhappiness, dissatisfaction, anxiety and depression than non-victims.

## 4. Model

**Figure 1:**
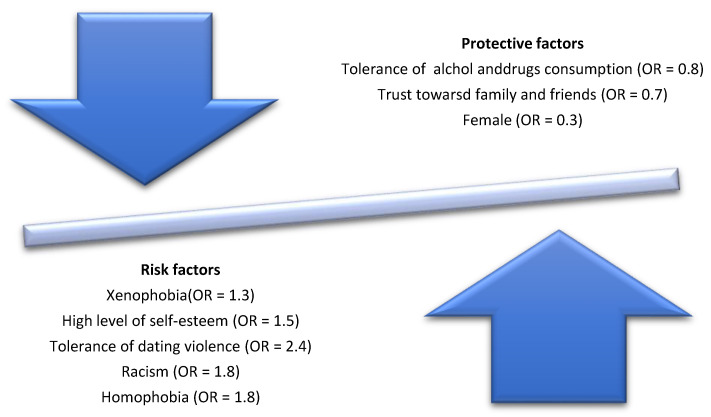
Risk and protection factors linked to bullying and cyberbullying as result of a binary logistic regression analysis.

As it was mentioned in the methodology paragraph, a binary logistic model with a stepwise forward algorithm has been applied to better understand the relation between the characteristics declared by respondents and the risk of belonging to the category of subjects at risk of involvement in bullying and cyberbullying. The result is a selection of those variables which were statistically significant during the data analysis. Figure 1 shows the significant variables with the OR value in parentheses. Results show that with a low level of tolerance towards foreigners the probability of belonging to the category at risk of involvement in bullying and cyberbullying increases by more than a third. In addition, this relation also applies to tolerance of dating violence (there is more than twice of probability of belonging to the category at risk), racism and homophobia (there is almost twice of probability of belonging to the category at risk). Finally, respondents with high levels of self-esteem are 50% more likely to belong to the category at risk compared to their peers. On the other hand, protective factors are related to low levels of tolerance of alcohol and drug consumption, high levels of trust towards parents, siblings and friends and, to a lesser extent, being female.

## 5. Discussion 

Bullying and cyberbullying phenomena need great attention because they are linked to a multiplicity of variables which can be both cause and effect of violent behaviours. Due to the complexity of these phenomena, the study attempted to provide a holistic approach in identifying risk and protective factors related to the involvement in bullying and cyberbullying. In fact, we believe that only by detecting the variables related to these problems within the same study a complete analysis of the adolescent reality can be carried out. In these kind of studies, social and individual plans should always be considered simultaneously. With the dual aim to avoid interpretations based on a single disciplinary level and to identify the strongest relationships between the variables used, we have analysed the influence of multiple factors on the involvement in bullying and cyberbullying. First of all, to estimate the spread of these phenomena within the school context we have directly identified victims of violent behaviours. Furthermore, in order to avoid the risk of underestimation of violence perpetrators due to the social desirability bias we have chosen not to submit a direct question to students. However, we have built a tolerance indicator identifying adolescents who are at risk to be involved in bullying and cyberbullying, as victims, perpetrators and bystanders. We have taken into account the family background of respondents, in terms of parental cultural status, considering the well-known influence of primary socialisation on youthful attitudes and behaviours. We have also analysed the set of social values of adolescents, with a focus on how these values influence and are reshaped within the peer group. Accordingly, attitudes and behaviours have been analysed through a set of indicators that measure the levels of tolerance towards violent and discriminatory behaviours, the propensity to consume of alcohol and drugs, the presence of social stereotype and the levels of trust towards family and friends. From a psychological perspective, the self-esteem of respondents was measured, as this variable is considered crucial for the involvement on violent behaviours. Using so many variables simultaneously could be a problem at an operational level. The questionnaire through which data were collected is a complex tool composed of many questions. This tool is the result of a specific methodological process based on several experiences that the authors have gained in recent years. The presence of a researcher in all classrooms where the questionnaire was submitted implied high research efforts and costs. However, it offered the opportunity to attend the compilation phase of the questionnaire and to answer any doubts of the students. This methodology has allowed us to work with a large number of psychosocial variables and to collect reliable data. The results of the study confirm many theories concerning the state of the art of the observed phenomena, adding further considerations on the characteristics of these problems. Bivariate statistical analysis confirmed the existence of a direct relation between tolerance of bullying and cyberbullying and social stereotyping. Indeed, to higher levels of adherence to stereotypes corresponds a greater tolerance of violent phenomena. Therefore, Traduzione di Inglese. results confirm close links between stigma and deviant attitudes. The influence of social values on the involvement in bullying and cyberbullying has been corroborated. Democracy, honesty, social participation and culture have been identified as protective values. The direct relation between low levels of trust (both towards family and teachers) and tolerance to bullying and cyberbullying has been confirmed. Moreover, higher levels of unhappiness among adolescents who are victims of violence have been confirmed. Finally, the relation between the involvement in bullying and cyberbullying and high levels of self-esteem among adolescents has been found. In particular, a direct relationship between victims of bullying and cyberbullying and low levels of self-esteem has been confirmed. In addition to these findings, the study identified a direct relation between low levels of recognition of violence and tolerance towards bullying and cyberbullying. An ad hoc logistics model has been developed in order to identify the strongest relations among the characteristics owned by respondents and the risk of involvement in bullying and cyberbullying. This model was built using the most significant variables resulted from the data bivariate analysis. 

## 6. Conclusions

The findings highlight specific risk and protective factors concerning the involvement of bullying and cyberbullying of Italian students. These factors are strongly related to social and cultural spheres. This result should not be considered as the primacy of sociological variables in the interpretation of these phenomenon of violence. However, it highlights the great influence on violent behaviours of social conditioning such as stereotypes, prejudice and fear of social and gender diversity. These factors produce racism, xenophobia and homophobia. The significant protective factors are low levels of tolerance of the consumption of alcohol and drugs, high levels of trust in friends and family and being female. On the one hand, these findings underline the need of carrying out other qualitative-quantitative researches to confirm, deepen and extend these interpretations. On the other hand, results already provide an operative indication to stem the phenomena of adolescent violence and deviance. This aspect seems to acquire greater importance in these times. In fact, as a result of the COVID-19 pandemic, an increase in internet and social media use, the risk of hyperconnection, internet addiction and on-line violent phenomena such as cyberbullying have been measured [51]. Strengthening the educational role of schools, as a secondary socialization agency, and support social and civic values of adolescents and families seems to be a precondition to struggle against violence. This is necessary to overcome fear and intolerance towards diversity, which is a prelude to discrimination and violence.

## Data Availability

The strictly anonymous database is kept in the CNR machines, is available to the research group and is not accessible from the outside. The research group undertakes to publish the research results in an accessible manner and to provide specific details of interest for the advancement of knowledge on young people and for social policies to public administrators and media.
